# Emulsification of Rosemary and Oregano Aqueous Extracts and Their In Vitro Bioavailability

**DOI:** 10.3390/plants11233372

**Published:** 2022-12-04

**Authors:** Sara Sirovec, Ana Jurinjak Tušek, Maja Benković, Davor Valinger, Tea Sokač Cvetnić, Jasenka Gajdoš Kljusurić, Tamara Jurina

**Affiliations:** Faculty of Food Technology and Biotechnology, University of Zagreb, Pierottijeva 6, 10000 Zagreb, Croatia

**Keywords:** rosemary and oregano aqueous extracts, phenolic compounds, bioavailability, emulsification

## Abstract

Due to their richness in phenolic compounds, Mediterranean plants such as rosemary and oregano are increasingly recommended for consumption for their numerous health benefits. The pH shift and the presence of digestive enzymes significantly reduce the bioavailability of these biochemicals as they pass through the gastrointestinal tract. To prevent this degradation of phenolic compounds, methods such as emulsification of plant aqueous extracts are used. The aim of this study was to investigate the effects of emulsification conditions on the chemical properties (total polyphenolic content and antioxidant activity) of emulsified rosemary and oregano extracts. Response surface methodology was applied to optimize sunflower oil concentration, rotational speed, and emulsifier concentration (commercial pea protein). The emulsions prepared under optimal conditions were then used in bioavailability studies (in vitro digestion). The antioxidant activity of the emulsified rosemary/oregano extracts, measured by the DPPH method, remained largely stable when simulating in vitro digestion. Analysis of antioxidant activity after in vitro simulation of the gastrointestinal system revealed a higher degree of maintenance (up to 76%) for emulsified plant extracts compared to aqueous plant extracts. This article contributes to our understanding of how plant extracts are prepared to preserve their biological activity and their application in the food industry.

## 1. Introduction

Recently, the market has become more interested in aromatic herbs from the Mediterranean region, such as rosemary and oregano, due to their high content of bioactive compounds and consequent medicinal properties [1]. These plants have been used since ancient times as spices in the traditional Mediterranean diet and as medicines in folk medicine. Nowadays, they are considered important factors for food safety and quality because they inhibit various factors that affect food quality, such as oxidation and microbial spoilage [2,3].

Oregano (*Origanum vulgare* L.) and rosemary (*Rosmarinus officinalis* L.) have been the subject of numerous studies due to their significant antioxidant potential and their role in the prevention of diseases related to oxidative stress [4,5,6,7]. Various factors such as environmental conditions, food processing, storage, and digestive system conditions reduce polyphenol concentrations and result in a reduction or complete loss of their bioactivity [8]. In addition, many polyphenolic compounds from natural sources have limited water solubility and unpleasant taste [9]. Therefore, the application of phenolic compounds requires a protective formulation, such as encapsulation [9]. Emulsions are considered the most popular encapsulation system due to their high encapsulation efficiency, preservation of chemical stability of the encapsulated molecules, and controlled release of molecules [10]. For example, Tian et al. [11] presented the protective effect of emulsification with a xanthan-locustane gum mixture on tea polyphenols, while Ye et al. [12] prepared thermodynamically stable water-in-oil emulsions loaded with tea polyphenols using zein as a stabilizer. Bamba et al. [13] efficiently encapsulated polyphenols and anthocyanins from blueberry pomace stabilized by whey proteins. All the mentioned studies described how emulsion stability depends on emulsion composition, homogenization conditions, and the type of emulsifier. Proteins, phospholipids, and other emulsifiers of natural or synthetic origin are used in foods [14]. The proteins that are currently most commonly used in the food industry are usually derived from milk, soy, eggs, etc. [15]. However, in recent years, there has been an increased interest in finding other protein sources that can be used as emulsifiers in the food industry. For this reason, interest in pea proteins as an alternative to soy proteins has increased in recent years due to their favorable amino acid profile, low allergenic potential, and high availability [16]. Although they are less soluble in water, studies show that they can be used as emulsifiers for the encapsulation and protection of lipophilic components [17,18].

Very little is known about the changes in the structural properties of polyphenols as they pass through the gastrointestinal tract [19,20,21]. After being taken into the mouth, these components are mixed with various salivary enzymes, undergo changes in pH and ionic strength, and then enter the gastrointestinal tract by peristaltic movement. Once in the stomach, polyphenols are subjected to pH 2 and mixed with digestive juices containing surfactants, phospholipids, proteins, proteolytic and lipolytic enzymes, and minerals. Only a small portion of phenolic acids are absorbed [22]. Modified polyphenols further migrate through the small intestine to the duodenum, where they are mixed with bile acids and pancreatic secretions. All these interactions are very complex and affect the bioavailability of bioactive compounds during the digestion process [19,20,21]. Only 5% of dietary polyphenols are absorbed in the small intestine, while the other 95% pass through the colon and are fermented by the microbiome [23]. In this context, in vitro methods have been developed to evaluate the bioavailability of biochemicals possessing antioxidant activity [24,25]. Simulation of in vitro digestion usually involves the decomposition of foods in the mouth, stomach, and small intestine, taking into account the presence of digestive enzymes and their concentration, pH, degradation time, and corresponding salts [26].

Due to the growing interest in using medicinal plant extract formulations, it is important to define the optimal processing conditions for their preparation. To the best of our knowledge, this is the first time that the influence of emulsifier concentration (commercial pea protein), rotational speed, and oil content on the stability of prepared oil-in-water emulsions containing rosemary/oregano extract has been studied. The physical (conductivity, zeta potential) and chemical (total polyphenolic content, antioxidant activity) properties of the prepared emulsions were analyzed. In addition, an in vitro simulation of the digestion process was performed to investigate the effectiveness of the emulsification process and in which form the mentioned plants should be best consumed to maintain human health. Thus, this article expands our knowledge of how plant extracts are processed to maintain their biological activity and how they are used in the food industry.

## 2. Results and Discussion

### 2.1. The Average Feret Diameters of Emulsified Rosemary/Oregano Extracts 

**Figure 1:**
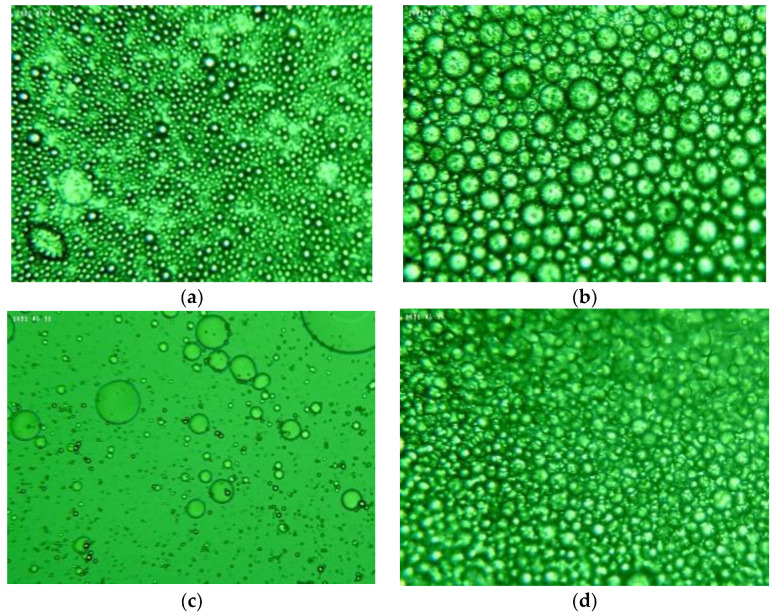
Microscopic images of oil-in-water emulsions containing oregano extract—(**a**) exp. No. 7; (**b**) exp. No. 9—and oil-in-water emulsions containing rosemary extract (**c**) exp. No. 9; (**d**) exp. No. 10.

According to Katsouli et al. [27], the particle size of the dispersed phase plays an important role in determining emulsion stability during storage; the stability and thus the shelf life of emulsions is increased by reducing their particle size. Droplets with a smaller size resist gravity separation [28]. After the emulsification process was completed, the emulsified rosemary/oregano extracts were photographed under an optical microscope (Figure 1), and the size of the emulsion droplets was determined using ImageJ software. ImageJ is a useful tool for quantitative image analysis in microscopy and is widely used in various scientific fields such as biology, medicine, and astronomy [29].

**Figure 2:**
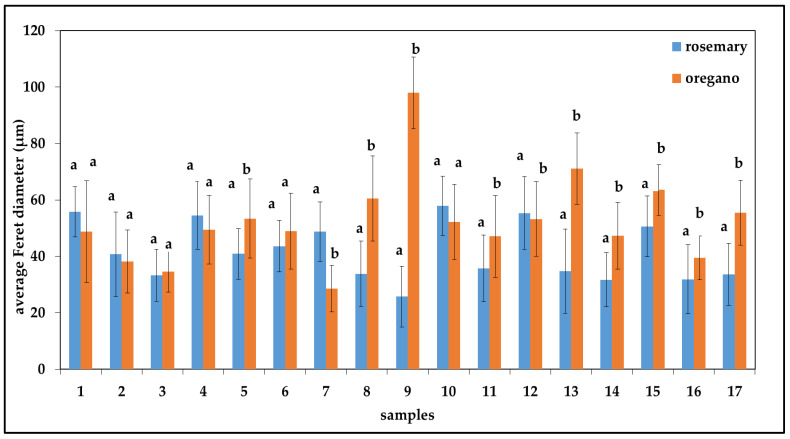
Average Feret diameters of oil-in-water emulsions containing rosemary extract and oil-in-water emulsions containing oregano extract. Different letters above the columns represent significant differences at *p* < 0.05.

For the emulsified oregano extracts (Figure 2), the highest average Feret diameter (97.97 µm) was determined for sample No. 9, while the lowest average Feret diameter was determined for sample No. 7 (28.56 µm). For emulsified oregano extracts, the lowest values of average Feret’s diameter were obtained for emulsions containing 1% commercial pea protein, while the highest average Feret’s diameter values were observed for emulsions containing 0.1% emulsifier concentration. Karadag et al. [30] and Sohn et al. [31] also reported a decrease in an emulsion droplet size with increasing emulsifier concentration. According to Sohn et al. [31], higher emulsifier concentration provides greater surface coverage of oil droplets, resulting in a decrease in the emulsion droplet size. For the emulsified rosemary extracts (Figure 2), the highest average Feret diameter (57.96 µm) was determined for sample No. 10, while the lowest average Feret diameter was determined for sample No. 9 (25.73 µm). For emulsified rosemary extracts with emulsifier concentrations of 0.1% and 0.5%, the lowest average Feret’s diameter values were measured. From the obtained results, it could be concluded that emulsified oregano extracts had slightly higher average Feret diameter values compared to emulsified rosemary extracts.

### 2.2. Physical and Chemical Properties of Oil-in-Water Emulsions Containing Rosemary/Oregano Extract 

Due to the growing interest in using medicinal plant extract formulations, it is necessary to determine the optimal process conditions for their preparation. In this work, the stability of prepared oil-in-water emulsions of rosemary/oregano extracts with the addition of commercial pea protein as an emulsifier was studied, and the effects of emulsifier concentration, rotational speed, and amount of oil in the emulsion were discussed. Emulsification of aqueous plant extracts was carried out based on the Box–Behnken experimental design. The physical and chemical properties of the obtained emulsified extracts are listed in Table 1 and Table 2. The actual pea protein concentrations for rosemary and oregano extracts were determined using the Bradford method before the emulsification process began. For 0.1%, 0.5% and 1% pea protein suspensions in rosemary extract, the values were 74.91 mg L^−1^, 484.81 mg L^−1^ and 767.49 mg L^−1^, respectively, while for 0.1%, 0.5% and 1% pea protein suspensions in oregano extract, the values were 279.86 mg L^−1^, 682.69 mg L^−1,^ and 1283.39 mg L^−1^, respectively.

Zeta potential or electrokinetic potential is a parameter that describes the electrochemical equilibrium on a solid surface and its liquid medium. It is of practical importance in controlling colloidal stability and flocculation processes. In the literature, emulsions with measured zeta potential values lower than −30 mV or higher than +30 mV are considered stable [32]. The higher the zeta potential, the stronger the electrostatic repulsion and the more stable the system [33], indicating the applicability of the zeta potential in predicting and controlling emulsion stability [34]. The measured zeta potential and conductivity values for the emulsified extracts are listed in Table 1. According to the results obtained for the emulsion of oil in aqueous rosemary extract, sample No. 8 (oil concentration 25%; emulsifier concentration 0.5%; rotational speed 35,000 rpm) had the lowest zeta potential value (−67.22 ± 2.24 mV), indicating the highest stability of the prepared oil-in-water emulsion containing rosemary extract. Sample No. 12 (oil concentration 15%; emulsifier concentration 1%; rotational speed 35,000 rpm) has the highest zeta potential value (−38.52 ± 3.28 mV), indicating the lowest stability of the prepared oil-in-water emulsion with rosemary extract. The zeta potential of the emulsions is affected by both the concentration of the vegetable oil and the concentration of the surfactant. Based on the present results, it can be concluded that an increase in the oil concentration increases the zeta potential, indicating that the fatty acids present in the oil have an important influence on the zeta potential. The results obtained are in agreement with those of Rezvani et al. [35], where increasing the ratio of oil phase to water phase significantly increased the zeta potential of an orange oil-in-water emulsion. For oil-in-water emulsions containing oregano extract, the lowest zeta potential value was also measured for sample No. 8 (−57.18 ± 4.47 mV), indicating the highest stability of the emulsified oregano extract, while sample No. 7 (oil concentration 10%; emulsifier concentration 0.5%; rotational speed 35,000 rpm) showed the lowest stability (−37.71 ± 2.31 mV). The zeta potential range measured for all 17 experiments indicates the stability of all prepared emulsified rosemary and oregano extracts (values below −30 mV). These results are in agreement with the results obtained by Hinderink et al. [36], who measured the zeta potential of emulsions containing 1% purified pea protein. The results showed negative zeta potential values or less than −30 mV even after two weeks, indicating the stability of the prepared emulsions. Oil-in-water emulsions with rosemary/oregano extract prepared with a natural emulsifier (commercial pea protein) in this work showed higher stability than oil-in-water emulsions with mint extract prepared with commercial emulsifiers (polyethylene glycol 1500, 6000, and 20,000). The zeta potential values of the emulsions prepared with polyethylene glycol of different molecular masses did not exceed values less than −32.128 mV [33]. Conductivity values (Table 1), measured for emulsified rosemary extracts, ranged from 0.0043 ± 0.00 mS cm^−1^ (sample No. 11: oil concentration 15%; emulsifier concentration 0.1%; rotational speed 35,000 rpm) to 0.0453 ± 0.05 mS cm^−1^ (sample No. 8) while for emulsified oregano extracts, ranged from 0.0051 ± 0.00 mS cm^−1^ (sample No. 1: oil concentration 10%; emulsifier concentration 0.1%; rotational speed 25,000 rpm) to 0.0499 ± 0.05 mS cm^−1^ (sample No. 6: oil concentration 25%; emulsifier concentration 0.5%; rotational speed 15,000 rpm). The highest conductivity value was obtained for emulsified oregano extract (sample No. 6: 0.0499 ± 0.05 mS cm^−1^) and emulsified rosemary extract (sample No. 8: 0.0453 ± 0.05 mS cm^−1^). 

Total polyphenolic content in the prepared emulsions was determined by the Folin–Ciocalteu method, while antioxidant activity was determined by the DPPH and FRAP methods (Table 2). The highest TPC (53.93 ± 0.76 mg_GAE_ g_dw_^−1^) was measured for the emulsified rosemary extract marked as sample No. 10 (oil concentration 15%; emulsifier concentration 1%; rotational speed 15,000 rpm), while the lowest TPC (1.35 ± 1.14 mg_GAE_ g_dw_^−1^) was observed for sample No. 2 (oil concentration 25%; emulsifier concentration 0.1%; rotational speed 25,000 rpm). For the emulsified oregano extracts, the highest TPC (113.79 ± 1.90 mg_GAE_ g_dw_^−1^) was measured for sample No. 3 (oil concentration 10%; emulsifier concentration 1%; rotational speed 25,000 rpm), while the lowest TPC (6.68 ± 0.00 mg_GAE_ g_dw_^−1^) was measured for sample No. 11. Vallverdù-Queralt et al. [37] studied the polyphenolic profile of the most commonly used herbs as spices and reported the highest TPC values for rosemary, thyme, and oregano. The results of this study showed that the emulsified oregano extracts had higher TPC values than the emulsified rosemary extracts. According to the USDA National Nutrient Database [38], fresh rosemary leaves (nutritional values per 100 g) contain 21.8 mg of vitamin C and 3.31 g of protein, while dried oregano leaves (values per 100 g) contain 2.3 mg of vitamin C and 9 g of protein. The differences in TPC values of the emulsified extracts could be due to the compounds such as ascorbic acid and proteins reacting with Folin–Ciocalteu reagent and interfering with the determination of TPC by the Folin –Ciocalteu assay [39,40]. Phenolic compounds in spicy vegetables are responsible for their antioxidant properties [41]. According to the results presented in Table 2, the highest antioxidant activity value determined by the DPPH method (0.35 ± 0.04 mmol_Trolox_ g_dw_^−1^) was obtained for the emulsified rosemary extract No. 12, while the lowest DPPH value (0.06 ± 0.01 mmol_Trolox_ g_dw_^−1^) was obtained for sample No. 11. Emulsified oregano extracts showed slightly higher DPPH values, compared to emulsified rosemary extracts. The highest DPPH value (0.83 ± 0.06 mmol_Trolox_ g_dw_^−1^) was observed for emulsified oregano extract No. 8, while the lowest value (0.30 ± 0.03 mmol_Trolox_ g_dw_^−1^) was found for sample No. 6. The highest FRAP value (0.49 ± 0.03 mmol_FeSO4·7H2O_ g_dw_^−1^) was obtained for the emulsified rosemary extract, marked as sample No. 3, while the lowest FRAP value was recorded for sample No. 9 (oil concentration 15%; emulsifier concentration 0.1%; rotational speed 15,000 rpm). For emulsified oregano extracts, the highest FRAP (1.01 ± 0.08 mmol_FeSO4·7H2O_ g_dw_^−1^) was measured for sample No. 10, while the lowest FRAP value (0.06 ± 0.01 mmol_FeSO4·7H2O_ g_dw_^−1^) was obtained for sample No. 1. As for the antioxidant activity measured by DPPH method, the emulsified oregano extracts showed higher antioxidant activity measured by the FRAP method than the emulsified rosemary extracts. The antioxidant activity of the emulsified plant extracts depended on both the polyphenolic content of the aqueous extracts used [42] and the phenolic content of the oil phase used [43,44]. As noted by Zeb [45] the sunflower oil used for emulsification in this work is rich in linoleic acid and oleic acid as well as various antioxidants, including phenolic molecules. The positive effect of the oil phase on the antioxidant activity of the prepared elusions was also described by Thanonkaew et al. [46]. They analyzed the effect of the concentration of cold press rice bran oil (CPRBO) in emulsions and showed that increasing the oil concentration in the emulsion increased the antioxidant activity.

### 2.3. Response Surface Modeling of the Emulsification Process

**Figure 3:**
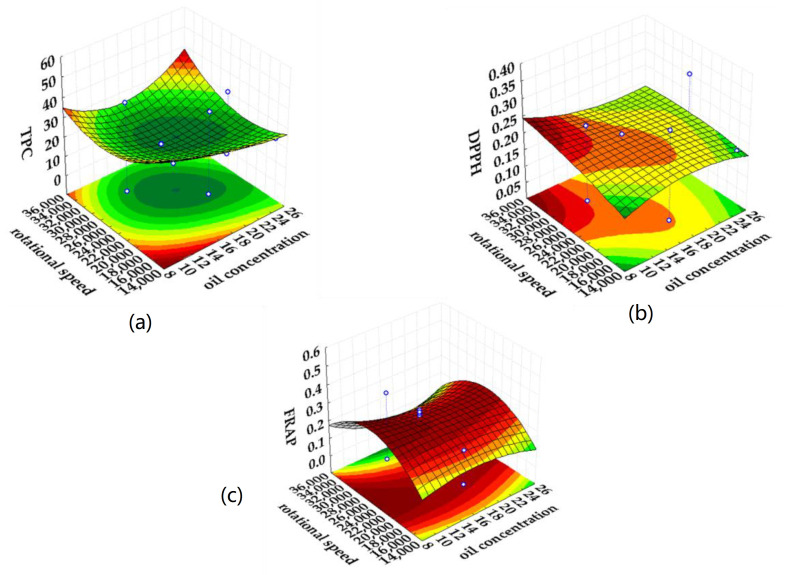
Response surfaces of the significant variables influencing the (**a**) the total polyphenolic content (TPC), antioxidant activity determined by (**b**) the DPPH and (**c**) the FRAP for the emulsified rosemary extract.

**Figure 4:**
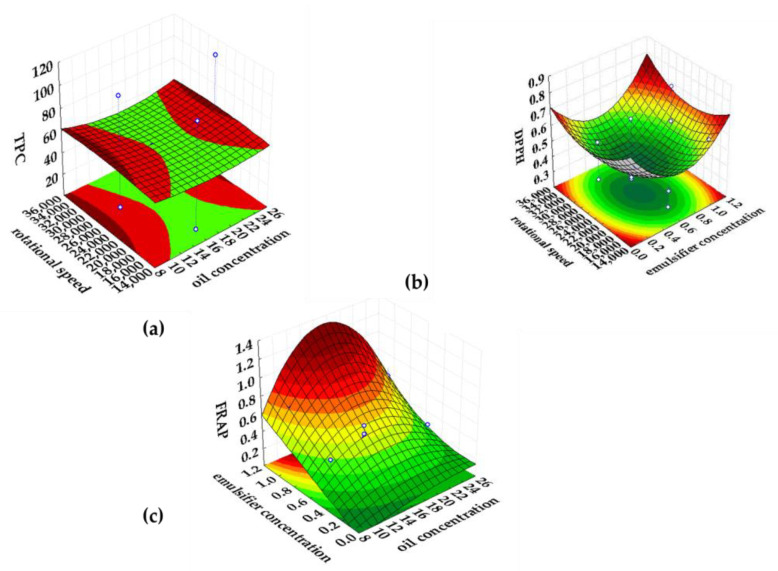
Response surfaces of the significant variables influencing (**a**) the total polyphenolic content (TPC), antioxidant activity determined by (**b**) the DPPH and (**c**) the FRAP for the emulsified oregano extract.

Second-degree polynomials with interacting members were used to describe the experimental data. The estimated model parameters and results from analysis of variance (ANOVA) are shown in Table 3 and Table 4, respectively, while the response surfaces of the significant variables affecting TPC, DPPH, and FRAP for both emulsified plant extracts are shown in Figure 3 and Figure 4, respectively. ANOVA was applied to investigate the effects of process variables on the chemical properties of oil-in-water emulsions containing rosemary/oregano extract. The regression parameters are presented together with the coefficients of determination in Table 3 and Table 4. For the emulsified rosemary extracts (Table 3), the emulsifier concentration and the rotational speed had a significant effect (*p* < 0.05) on the TPC. The obtained positive value for the linear parameter indicates that an increase in the commercial pea protein concentration causes an increase in the TPC value for emulsified rosemary extracts. A negative value for the linear parameter indicates that an increase in rotational speed results in a decrease in the TPC value of the emulsions. Oil concentration had no statistically significant effect on the TPC value (*p* > 0.05). From the calculated coefficient of determination (R^2^ = 0.94; R^2^_adj._ = 0.90), it can be concluded that the influence of the process parameters on the TPC value can be excellently described by the developed predictive models.

Regarding the antioxidant activity determined by the DPPH method, the influence of the process parameters on the DPPH value of the emulsified rosemary extracts cannot be described by the prediction models developed (R^2^ = 0.35; R^2^_adj_. = 0.00). The studied process parameters had no significant effect (*p* > 0.05) on the DPPH values determined in the experiment. Regarding the antioxidant activity determined by the FRAP method, an increase in oil concentration and rotational speed leads to a decrease in the FRAP value of the emulsified rosemary extracts, while an increase in emulsifier concentration leads to an increase in the FRAP values. This can be explained by the antioxidant potential of the emulsifier used. This means that a higher concentration of the emulsifier leads to a higher antioxidant activity of the prepared emulsion. Based on the positive value obtained for the quadratic parameter, the rotational speed had a statistically significant effect (*p* < 0.05) on the FRAP, compared to the oil and emulsifier concentration (*p* < 0.05). The calculated coefficient of determination (R^2^ = 0.76; R^2^_adj._ = 0.62) indicates a good relationship between the FRAP and the process variables.

For the oil-in-water emulsions containing oregano extract (Table 4), the studied process parameters had a significant effect (*p* < 0.05) on the obtained TPC values. An increase in the emulsifier concentration leads to a significant increase in the TPC value of the emulsified oregano extracts (the largest positive value of the linear regression coefficient was predicted). Based on the calculated coefficient of determination (R^2^ = 0.99; R^2^_adj._ = 0.99), it can be concluded that the influence of the process parameters on the TPC value of the emulsions can be excellently described by the developed predictive models. The concentration of commercial pea protein and the rotational speed had a significant influence on the DPPH value of the emulsified oregano extracts (*p* < 0.05) compared to the influence of oil concentration (*p* > 0.05). From the positive values of the linear regression coefficients, it can be seen that an increase in emulsifier concentration and rotational speed leads to an increase in the DPPH value of the emulsions. According to the coefficient of determination (R^2^ = 0.76; R^2^_adj._ = 0.60), there is a strong relationship between the experimental DPPH values and the parameters of the emulsification process. Regarding the antioxidant activity determined by the FRAP method, emulsifier and oil concentration significantly influenced the FRAP value of emulsified oregano extracts (*p* < 0.05) compared to the influence of rotational speed (*p* > 0.05). Increasing the concentration of the commercial pea protein led to an increase in the FRAP value of the emulsified extracts. The determination coefficient calculated for the FRAP value (R^2^ = 0.91; R^2^_adj._ = 0.84) indicates a strong relationship between FRAP and the process parameters, which means that the predictive models excellently describe the experimental FRAP values of the emulsified oregano extracts.

### 2.4. Optimization of the Emulsification Process with Respect to Chemical Properties of Oil-in-Water Emulsions Containing Rosemary/Oregano Extract with the Addition of Emulsifier

Response Surface Methodology (RSM) was applied to define optimal conditions for the preparation of oil-in-water emulsions containing rosemary/oregano extract with the addition of commercial pea protein as an emulsifier. The influence of three independent variables (process parameters) such as oil concentration in emulsified plant extracts, emulsifier concentration, and rotational speed was analyzed at three levels: (oil concentration = 10%, 15%, 25%; emulsifier concentration = 0.1%, 0.5%, 1%; rotational speed = 15,000 rpm, 25,000 rpm, 35,000 rpm). The duration of emulsification was 4 min. Optimization was performed simultaneously for the chemical properties of the emulsions based on the desirability profiles obtained from the RSM-predicted values. The desirability scale in the range of 0 (undesirable) to 1 (very desirable) was used [47].

**Figure 5:**
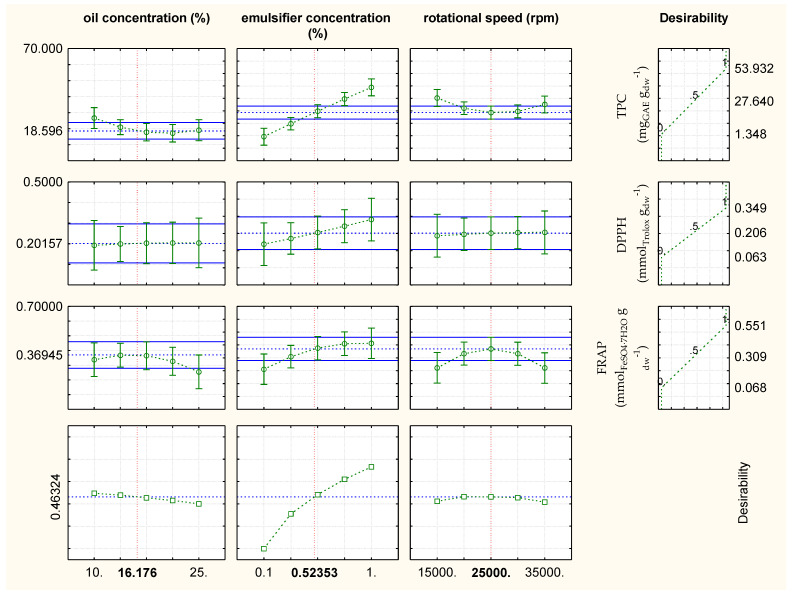
Results of the optimization of the emulsification conditions with regard to chemical properties (TPC, DPPH, FRAP) for oil-in-water emulsions containing rosemary extract.

Based on the obtained results from Figure 5, the optimal process conditions for the emulsification of plant extracts, concerning chemical properties, for the emulsified rosemary extracts, were: oil concentration = 16.17%, emulsifier concentration = 0.52% and rotational speed = 25,000 rpm with values of TPC = 18.59 mg_GAE_ g_dw_^−1^, DPPH = 0.2 mmol_Trolox_ g_dw_^−1^ and FRAP = 0.37 mmol_FeSO4·7H2O_ g_dw_^−1^. The optimal experimental value for DPPH was the same as the RSM-predicted value, while values for TPC and FRAP slightly differed from the RSM-predicted TPC and FRAP values. The model’s predicted values were: TPC = 27.64 mg_GAE_ g_dw_^−1^, DPPH = 0.2 mmol_Trolox_ g_dw_^−1^, and FRAP = 0.31 mmol_FeSO4·7H2O_ g_dw_^−1^.

**Figure 6:**
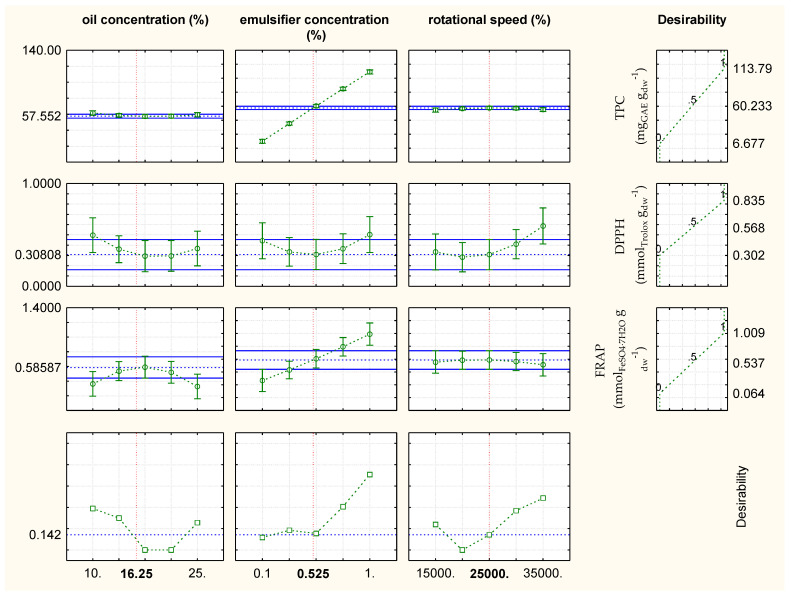
Results of the optimization of the emulsification conditions with regard to chemical properties (TPC, DPPH, FRAP) for oil-in-water emulsions containing oregano extract.

The optimal process conditions for the emulsified oregano extracts were as follows (Figure 6): oil concentration = 16.25%, emulsifier concentration = 0.52%, and rotational speed = 25,000 rpm with values of TPC = 57.55 mg_GAE_ g_dw_^−1^, DPPH = 0.31 mmol_Trolox_ g_dw_^−1^, and FRAP = 0.58 mmol_FeSO4·7H2O_ g_dw_^−1^. The optimal experimental values for TPC and FRAP were close to the RSM predicted values, while the optimal experimental value for DPPH significantly differed from the predicted value. Model predicted values were TPC = 60.23 mg_GAE_ g_dw_^−1^, DPPH = 0.57 mmol_Trolox_ g_dw_^−1^, and FRAP = 0.54 mmol_FeSO4·7H2O_ g_dw_^−1^.

Based on the obtained results for the optimization of emulsification, increasing the oil concentration up to 25% did not affect TPC and DPPH values of the emulsified rosemary extracts but significantly decreased FRAP values. TPC and DPPH values were considerably affected by increasing the commercial pea protein content from 0.1% to 1%; the relationship between TPC and DPPH values and emulsifier concentration is proportional. The emulsifier concentration had a minor impact on FRAP results. While having no impact on DPPH, increasing rotational speed from 15,000 rpm to 35,000 rpm enhanced TPC. From the minimum to maximum rotational speed, the FRAP value was significantly reduced (Figure 5).

Considering the emulsified oregano extracts (Figure 6), an increase in the oil concentration and rotational speed did not influence TPC, while the increase in the emulsifier concentration from 0.1% to 1% significantly increased TPC value. The increase in the oil concentration influenced the DPPH value, and the increase in the emulsifier concentration and rotational speed resulted in a significant DPPH increase. An increase in the oil concentration significantly decreased FRAP value, while an increase in the concentration of commercial pea protein significantly increased FRAP value. Rotational speed did not influence the FRAP value.

The investigations by Mehmood et al. [48] and Chong et al. [49] provided more evidence for the successful application of RSM in the assessment of parameters affecting emulsification. Mehmood and colleagues investigated the effects of olive oil concentrations, surfactant levels, and ultrasonic homogenization time on the physicochemical characteristics of generated olive-oil-based nanoemulsions for the encapsulation of α-tocopherol using a food-grade non-ionic surfactant. A significant influence of independent variables was observed on the response variables. They concluded that the particle size of nanoemulsions mainly depended on homogenization time, while surfactant concentration had a significant effect on antioxidant activity. Chong et al. [49] prepared nanoemulsions consisting of palm oil, rich in tocopherol, with the addition of commercial emulsifiers (Tween 80, Span 80). The effects of emulsifier concentration, solvent amount, and homogenization pressure were investigated toward droplet size and polydispersity index. The actual values of the nanoemulsion prepared at optimal conditions were in good agreement with the predicted values obtained from RSM.

### 2.5. The In Vitro Digestion of Oil-in-Water Emulsions Containing Rosemary/Oregano Extract 

The oil-in-water emulsions containing rosemary/oregano extract, prepared at the optimal conditions determined by the RSM were further used for in vitro simulation of the gastrointestinal system. After in vitro simulation, physical (TDS, conductivity) and chemical (TPC, DPPH, FRAP) properties of the plant extracts and emulsified plant extracts were measured. Before starting in vitro digestion, initial concentrations of the samples were obtained by measuring their physical and chemical properties (Table 5).

According to the definition, electrical conductivity is a measure of how easily an electric current can pass through water or some other solution or suspension. It is influenced by parameters such as the presence of weak electrolytes, pH, and temperature [26]. Conductivity could be used for determining the antioxidant activity of aqueous plant extracts [50,51]. According to the results presented in Table 5, initial conductivity and TDS values were higher for plant extracts compared to emulsions. The highest initial TDS and conductivity were measured for oregano extract (0.31 ± 0.01 g L^−1^ and 0.63 ± 0.00 mS cm^−1^) and rosemary extract (0.09 ± 0.00 g L^−1^ and 0.18 ± 0.00 mS cm^−1^) compared to emulsified oregano extract (0.04 ± 0.00 g L^−1^ and 0.01 ± 0.04 mS cm^−1^) and emulsified rosemary extract (0.02 ± 0.00 g L^−1^ and 0.04 ± 0.00 mS cm^−1^). 

**Figure 7:**
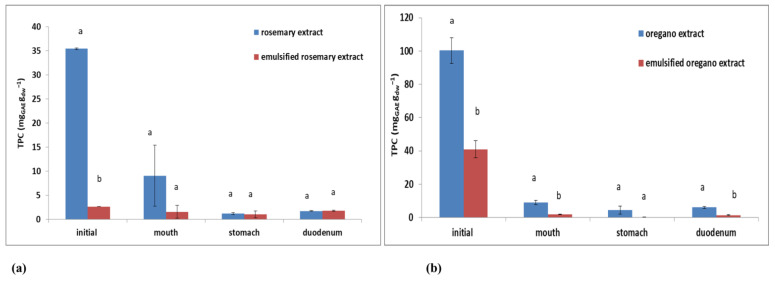
Changes in the total polyphenolic content (TPC) of plant extracts and oil-in-water emulsions containing extract during in vitro digestion for (**a**) rosemary and (**b**) oregano. Different letters above the columns represent significant differences at *p* < 0.05.

After in vitro digestion, TDS and conductivity of plant extracts and emulsions increased due to the addition of digestive enzymes, HCl, and bile salts throughout the whole simulation process of a gastrointestinal system [19] (Table 5). According to Figure 7, aqueous plant extracts had higher TPC values, concerning their emulsions, before in vitro simulation of gastrointestinal digestion. The initial TPC value for oregano extract was 100.27 ± 7.71 mg_GAE_ g_dw_^−1^, and for rosemary extract, the TPC value was 35.00 ± 0.19 mg_GAE_ g_dw_^−1^. The initial TPC value for emulsified oregano extract was 40.95 ± 5.19 mg_GAE_ g_dw_^−1^, while for the emulsified rosemary extract, the TPC value was 2.70 ± 0.00 mg_GAE_ g_dw_^−1^. The total polyphenolic content of aqueous plant extracts and emulsified plant extracts decreased during in vitro digestion. This TPC decrease was the most significant for the aqueous oregano extract and for the emulsified oregano extract (Figure 7b) compared to rosemary (Figure 7a). Regarding oregano extract and emulsified oregano extract, TPC decreased by approximately 95%, while for the emulsified rosemary extract, about 32% of TPC decreased. 

TPC values for plant extracts and emulsified plant extracts, after the third step of in vitro digestion (duodenum), increased compared to their values measured in the second step (stomach) (Figure 7). Tarko and Duda-Chodak [52] reported a higher polyphenol value and antioxidant activity after finished digestion due to the action of digestive enzymes and pH changes in the stomach and duodenum, leading to polyphenols decomposition to components with higher antioxidant potential.

**Figure 8:**
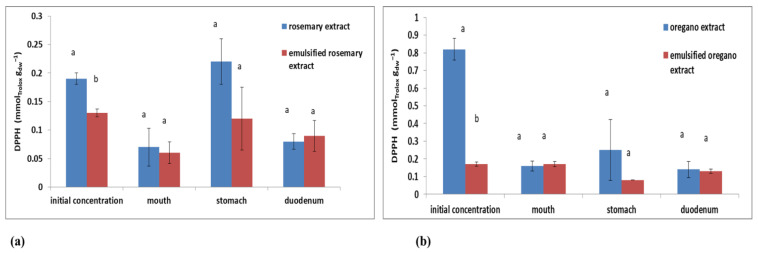
Changes in the antioxidant activity (DPPH) of plant extracts and oil-in-water emulsions containing extract during in vitro digestion for (**a**) rosemary and (**b**) oregano. Different letters above the columns represent significant differences at *p* < 0.05.

Results obtained for the antioxidant activity determined by the DPPH method showed higher initial values measured for plant extracts compared to emulsified plant extracts (Figure 8), which was expected concerning their initial TPC values (Figure 7). The highest DPPH decrease after the first digestion step was observed for oregano extract in comparison with its initial value (0.82 ± 0.06 mmol_Trolox_ g_dw_^−1^), while DPPH value measured for the emulsified oregano extract was completely preserved (0.17 ± 0.01 mmol_Trolox_ g_dw_^−1^). After the second digestion step, DPPH values started to increase, for all analyzed samples (Figure 8), as a result of pH influence and the difference in the ability of plants to reduce DPPH due to different portions of bioactive components [53]. The highest DPPH in the stomach was measured for oregano extract (0.25 ± 0.17 mmol_Trolox_ g_dw_^−1^). After the third step, DPPH values for all samples decreased, with the exception of emulsified oregano extract (Figure 8b).

Generally speaking, DPPH values measured for oregano extract and emulsified oregano extract were higher compared to rosemary extract and emulsified rosemary extract. It is evident that plant emulsions prepared under optimal conditions showed better preservation of the antioxidant activity determined by the DPPH method compared to their aqueous extracts. Results obtained for the antioxidant activity determined by the FRAP method were not in agreement with the results obtained by the DPPH method during in vitro digestion. The reason could be the different profiles of polyphenolic components present in plants that can affect the ability to reduce certain radicals [47]. The higher initial FRAP values were measured for plant extracts (0.61 ± 0.02 mmol_FeSO4·7H2O_ g_dw_^−1^ for rosemary and 1.38 ± 0.02 mmol_FeSO4·7H2O_ g_dw_^−1^ for oregano, respectively). The initial FRAP values measured for emulsified plant extracts were 0.21 ± 0.01 mmol_FeSO4·7H2O_ g_dw_^−1^ for rosemary and 0.56 ± 0.01 mmol_FeSO4·7H2O_ g_dw_^−1^ for oregano.

**Figure 9:**
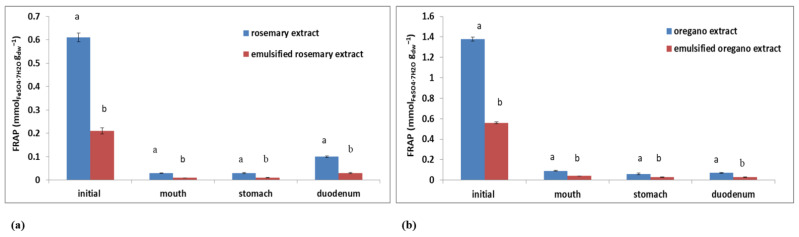
Changes in the antioxidant activity (FRAP) of plant extracts and oil-in-water emulsions containing extract during in vitro digestion for (**a**) rosemary and (**b**) oregano. Different letters above the columns represent significant differences at *p* < 0.05.

After the first digestion step (in the mouth), FRAP values significantly decreased for the rosemary extract and emulsified rosemary extract (Figure 9a). The same trend could also be observed for oregano samples (Figure 9b). After the second digestion step (in the stomach), FRAP values, for both extracts and emulsions, did not significantly change compared to the first step (Figure 9). At the end of in vitro simulation of gastrointestinal digestion (duodenum), the highest FRAP value was measured for rosemary extract (0.1 ± 0.00 mmol_FeSO4·7H2O_ g_dw_^−1^), while the lowest/same was measured for emulsified rosemary and oregano extracts (0.03 ± 0.00 mmol_FeSO4·7H2O_ g_dw_^−1^). The obtained results show that the antioxidant activity measured by the FRAP method remained mostly conserved in emulsified rosemary extract.

### 2.6. Bioavailability of Polyphenolic Compounds from Rosemary/Oregano Extracts and Oil-in-Water Emulsions Containing Rosemary/Oregano Extract

After in vitro simulation of the gastrointestinal system, the bioavailability of total phenolics from plant extracts and emulsified plant extracts was determined, and results are presented in Table 6. Bioavailability was calculated as the ratio of TPC (DPPH, FRAP) after the end of in vitro digestion (duodenum) and the initial concentration (before starting in vitro digestion).

Regarding TPC, the highest bioavailability was calculated for the emulsified rosemary extract (67.78%), while other samples show a significant decrease in TPC during in vitro digestion (Figure 7). The results indicate a significant influence of pH changes on phenolic compounds during in vitro digestion [26,54]. Gutiérrez-Grijalva et al. [54] pointed out that the phenolic compounds’ stability under various pH conditions, such as those present during gastrointestinal digestion, is influenced by the composition of the molecules and the distribution of the -OH radicals on their rings. They also stated that the loss of certain phenolic compounds following the GI process has been linked, in addition to pH changes, to potential interactions between polyphenols and other elements of the digestive fluids, such as enzymes and electrolytes, which requires additional research.

Regarding the antioxidant activity determined by the DPPH method, higher bioavailability values were observed for emulsified plant extracts compared to plant extracts (Table 6). This could be an indication that the desired effect of preserving antioxidant activity was achieved by the emulsification of plant extracts. The highest bioavailability was observed for emulsified oregano extract (76.47%), followed by emulsified rosemary extract (69.23%) and rosemary extract (42.11%). Results have shown that the antioxidant activity of emulsified plant extracts measured by the DPPH method remained mostly stable when simulating the in vitro digestion process (Figure 8). The bioavailability of the plant extracts and emulsified plant extracts for the FRAP method has shown significantly lower values compared to the DPPH values. Results have shown low preservation of the antioxidant activity determined by the FRAP method (Table 6, Figure 9) compared to the DPPH method.

## 3. Materials and Methods

### 3.1. Materials

#### 3.1.1. Plant Materials, Sunflower Oil and Emulsifier

Dried rosemary (*Rosmarinus officinalis* L.) and organic pea protein powder were purchased from Nutrigold (Zagreb, Croatia), and dried oregano (*Origanum vulgare* L.) was purchased from SonnentoR (Sprögnitz, Austria). Edible sunflower oil (VitaDor, Germany) was obtained from a local supermarket. Plant materials were collected during the season of 2020 (oregano) and 2021 (rosemary), dried naturally, and stored at ambient conditions before use. The country of origin of rosemary is India, and the country of origin of oregano is Austria.

#### 3.1.2. Chemicals 

TPTZ (2,4,6-tris(2-pyridyl)-s-triazine), gallic acid (98%), iron (II) sulphate heptahydrate, DPPH (1,1-diphenyl-2-picrylhydrazyl), Trolox (6-hydroxy-2,5,7,8-tetramethylchromane-2-carboxylic acid), and sodium chloride were obtained from Sigma-Aldrich Chemie (Steinheim, Germany). Hydrochloric acid (30%), iron (III) chloride hexahydrate, sodium carbonate, and sodium chloride were purchased from Gram-Mol d.o.o. (Zagreb, Croatia). Sodium acetate trihydrate was obtained from J.T. Baker (Deventer, The Netherlands). Sodium hydrogen carbonate was obtained from Franck (Zagreb, Croatia). Folin–Ciocalteu reagent, disodium hydrogen phosphate, and sodium dihydrogen phosphate dihydrate were obtained from Kemika d.d. (Zagreb, Croatia), acetic acid was purchased from T.T.T. d.o.o. (Sveta Nedjelja, Croatia), and methanol was obtained from Carlo Erba Reagents S.A.S. (France). A-amylase, pancreatin, and bile salts were purchased from Sigma-Aldrich (St. Louis, MO, USA), while pepsin was obtained from Fisher Scientific UK (Loughborough, UK). Chemicals were of analytical reagent grade.

### 3.2. Methods

#### 3.2.1. Preparation of Aqueous Plant Extracts

Dry rosemary/oregano (12 g) was mixed with 600 mL of deionized water, previously thermostated at 80 °C. Solid–liquid extraction was performed using Ika HBR4 digital oil-bath (IKA-Werk GmbH & Co.KG, Staufen, Germany) at *T* = 80 °C and 250 rpm for 30 min. After extraction, samples were filtered, using a vacuum filtration system, through a 100% cellulose paper filter (LLG Labware, Meckenheim, Germany) with a 5–13 µm diameter pore size [50]. Aqueous plant extracts were further used for the preparation of oil-in-water emulsions containing plant extract with the addition of an emulsifier (organic pea protein powder).

#### 3.2.2. Preparation of Pea Protein Suspensions in Aqueous Plant Extracts

Pea protein suspensions in aqueous plant extracts were prepared according to Sridharan et al. [55] with a slight modification of their method. In order to prepare 1% pea protein concentration, 4.5 g of pea protein powder from controlled organic cultivation was suspended in the 450 mL of aqueous rosemary/oregano extracts. Suspension was homogenized under magnetic stirring (500 rpm) at room temperature for several minutes. The pH value was adjusted to 7 by addition of NaOH (0.5 M). After pH adjustment, pea protein suspension was filtered using a filter paper with a cut off of 4–12 μm (Rundfilter, MN 640 m dia 11 cm, Macherey-Nagel, Düren, Germany) using vacuum filtration. Preparation of 0.1% and 0.5% pea protein concentrations in aqueous rosemary/oregano extracts was performed by diluting 1% pea protein concentration with distilled water.

#### 3.2.3. Determination of the Concentration of Commercial Pea Protein According to the Bradford Method

The actual protein concentrations in 0.1%, 0.5%, and 1% pea protein suspensions were determined by the Bradford colorimetric method, using bovine serum albumin (BSA) as standard (1 mg mL^−1^) [56]. A 0.5 mL amount from each sample was mixed with 0.5 mL of Bradford reagent, and after 30 min incubation, the absorbance was measured at 595 nm using a UV–VIS spectrophotometer (Biochrom Libra S12, Cambridge, UK). Data were expressed as mg L^−1^.

#### 3.2.4. Oil-in-Water Emulsions Containing Plant Extract Preparation 

Oil-in-water emulsions containing rosemary/oregano extract were prepared according to the conditions defined using Box–Behnken experimental design [57] (Table 7). A certain volume of aqueous rosemary/oregano extracts containing an appropriate emulsifier concentration (organic pea protein) was placed in a 15 mL falcon test tube with a certain volume of sunflower oil, in order to prepare 7 mL of emulsion. The mixture was homogenized using a homogenizer (OMNI TH220-PCRH homogenizator, Omni International, USA) for 4 min. The zeta potential and electrical conductivity of the prepared emulsions were measured immediately, while the rest of the samples were stored at 4 °C until further analysis for chemical properties (total polyphenolic content, antioxidant activity).

After the optimal conditions for the emulsification of aqueous plant extracts were determined, the same optimal conditions were used for preparation of oil-in-water emulsions containing rosemary/oregano extract, which were further used for in vitro simulation of the gastrointestinal system (GI).

#### 3.2.5. Extraction Yield

Extraction yield was expressed as the dry matter content of the plant material. Dry matter was determined using a standard AOAC method 930.15 [58]. A certain amount of plant material samples (1–5 ± 0.0001 g) was dried in weighing dishes to constant weight at 105 °C, for 3 h. All measurements were performed in duplicate.

#### 3.2.6. Determination of Physical Characteristics of Oil-in-Water Emulsions Containing Plant Extract 

##### Determination of Zeta Potential, Electrical Conductivity and Total Dissolved Solids

The surface charges of the emulsion droplets and electrical conductivity were measured using a Zetasizer Ultra instrument (Malvern Panalytical Limited ZSU3305, Malvern, UK). The emulsion samples were diluted two hundred times in distilled water. The diluted samples were then filled in a Malvern omega cuvette. The samples were placed in the instrument and equilibrated for 10 min before measurements were performed. All measurements were performed in triplicate.

Total dissolved solids (TDS) were determined using the SevenCompact conductometer (SevenCompact, Mettler Toledo, Switzerland). The probe was immersed into the sample (aqueous plant extracts or oil-in-water emulsions containing plant extract), and the values were read. Samples were homogenized before each measurement. All measurements were performed in duplicate. 

##### Determination of the Average Feret’s Droplet Diameter of Oil-in-Water Emulsions Containing Rosemary/Oregano Extract 

According to Grgić et al. [59], Feret diameter is defined as the perpendicular distance between two tangents located on opposite sides of a particle. Samples of oil-in-water emulsions containing rosemary/oregano extract were photographed using a microscope equipped with a camera (BTC type LCD-35, Bresser, Germany) at 10x magnification. The average Feret diameter of the droplets was measured using the software tool ImageJ (v.1.8.0. National Institutes of Health, Bethesda, MD, USA). A total of 30 droplets were analyzed on each image. The average Feret’s diameter was estimated as the average value of 30 measurements of the Feret’s droplet diameter [59]. 

##### In Vitro Digestion

The in vitro digestion of aqueous plant extracts and oil-in-water emulsions containing plant extract prepared at optimal conditions was performed according to the method described by Ortega et al. [60]. This method is based on mimicking the digestive process in the mouth, stomach (gastric digestion), and small intestines (duodenal digestion). The first step (mouth) includes the addition of 40 mg α-amylase in 40 mL phosphate buffer (pH = 6.9 with 0.04% NaCl and 0.004% CaCl_2_) to 4 mL of the aqueous plant extracts and emulsified extracts, respectively. The reaction mixture was shaken and incubated for 5 min at 37 °C. Before the second step (stomach), it is necessary to adjust the pH value of the reaction mixture to 2 by adding HCl (37%). After pH adjustment, the second step starts with the addition of 60 mg of porcin-pepsin solution in 4 mL HCl (0.01 mol L^−1^). This mixture was shaken and incubated at 37 °C for 2 h. Before duodenal digestion, the pH value of the reaction mixture was adjusted to 6.5 by adding NaHCO_3_. The third step starts with the addition of pancreatin (0.08 g pancreatin dissolved in 10 mL phosphate buffer) and bile salts (0.5 g bile salts dissolved in 10 mL phosphate buffer). The reaction mixture was shaken and incubated at 37 °C for 2 h. Samples (1 mL) were taken after each digestion phase and analyzed for electrical conductivity, TDS, TPC, and the antioxidant activity (DPPH, FRAP) [26]. 

#### 3.2.7. Determination of Chemical Characteristics of Aqueous Plant Extracts and Oil-in-Water Emulsions Containing Plant Extract 

##### Determination of Total Polyphenolic Content (TPC)

Total polyphenolic content (TPC) of aqueous rosemary/oregano extracts and oil-in-water emulsions containing rosemary/oregano extract was determined spectrophotometrically, according to Singleton and Rossi [61], using Folin–Ciocalteu reagent. Shortly, 7.9 mL of distilled water was mixed with 500 µL of Folin–Ciocalteu reagent (Folin–Ciocalteu reagent:water at a 1:2 ratio) and 100 µL sample. The reaction was started with addition of 1.5 mL of 20% Na_2_CO_3_ solution. After 2 h of incubation in a dark place, the absorbance of the reaction mixture was measured at *λ* = 765 nm using spectrophotometer (Biochrom Libra S11, Cambridge, UK). Measurements were performed in duplicates and the results were derived from a calibration curve for gallic acid (GA) (0–500 mg L^−1^) and expressed as mg GA equivalents (GAE)/g dry weight (dw) plant material [50].

##### Determination of Antioxidant Activity by DPPH Method

Antioxidant activity measured by DPPH (1,1-diphenyl-2-picrylhydrazyl) scavenging method was performed as described by Brand-Williams et al. [62]. The reaction mixture consisted of 100 μL sample and 3.9 mL of DPPH radical (0.094 M) dissolved in methanol. After 30 min of incubation, the absorbance of the reaction mixture was measured at *λ* = 515 nm. Measurements were performed in duplicate, and the results were derived from a calibration curve for Trolox (0–1 mmol L^−1^) and expressed as mmol Trolox equivalents/g dry weight (dw) plant material [50].

##### Determination of Antioxidant Activity by FRAP Method

The FRAP (Ferric ion reducing antioxidant power) assay was carried out according to Benzie and Strain [63]. The reaction mixture consisted of 50 μL sample and 950 μL of FRAP reagent. The mixture was incubated for 4 min, and the absorbance was measured at *λ* = 593 nm. Measurements were performed in duplicate, and the results were derived from a calibration curve for FeSO_4_ · 7H_2_O (0–1 mmol L^−1^) and expressed as mmol FeSO_4_ ⋅ 7H_2_O equivalents/g dry weight (dw) plant material [50].

#### 3.2.8. Response Surface Methodology (RSM)

Response Surface Methodology (RSM) was used to investigate the influence of three independent variables (oil concentration in emulsified plant extracts, emulsifier concentration, rotational speed) on the chemical characteristics (TPC, DPPH, FRAP) of emulsified extracts using software Statistica 14.0. (TIBCO^®^ Statistica, Palo Alto, California, USA). The experiment was performed according to Box–Behnken experimental design [59]. Each independent variable was analyzed at three levels: oil concentration (10%, 15%, 25%), emulsifier concentration (0.1%, 0.5%, 1%), and rotational speed (15,000 rpm, 25,000 rpm, 35,000 rpm). The resulting experimental design comprised 17 experiments, as shown in Table 7. For the description of the experimental data obtained for chemical properties (TPC, DPPH, FRAP) of oil-in-water emulsions containing rosemary/oregano extract, prediction models (second-order polynomial models) were developed. In order to describe how well models fit a set of experimental data, the coefficient of determination (R^2^) was calculated.

#### 3.2.9. Statistical Analysis

The means of the results of physical and chemical properties of oil-in-water emulsions containing rosemary/oregano extract with the addition of commercial pea protein as emulsifier and physical and chemical properties of rosemary/oregano extracts and oil-in-water emulsions containing rosemary/oregano extract, with the addition of commercial pea protein as emulsifier, before and during in vitro digestion, were evaluated using analysis of variance (ANOVA) while Tukey’s test was used to compare significant differences (*p* < 0.05) between the samples. The ANOVA was performed using Statistica 14.0. (TIBCO^®^ Statistica, Palo Alto, California, USA). Differences between different plant aqueous extracts/emulsions at the same experimental conditions were analyzed, as well as the differences between different experimental conditions for the selected plant aqueous extracts/emulsions. Prior to statistical analysis, the normality of the data was evaluated using Kolmogorov–Smirnov test, implemented in Statistica software. *p*-values of the analyzed data sets were not significant, and therefore the assumption of normality can be accepted. Homogeneity of variance was assessed using Levene’s test implemented in Statistica software. *p*-values of the analyzed data sets were higher than 0.05, indicating the homogeneity of the variances.

## 4. Conclusions

Phenolic compounds derived from plant sources have the potential to act as natural antioxidants and are relevant to food preservation and the prevention of human diseases. It is important to maintain the biological activity of these phytochemicals, and emulsification represents a good way of keeping their diverse biological effectiveness. To highlight the advantages of emulsion systems often used in the food industry, it is necessary to improve the process of emulsion preparation. The optimization of emulsification represents a good way to select corresponding process conditions in order to create emulsions with good nutritional, functional, and sensory properties, as well as biological activities. Additionally, the utilization of food-grade emulsions containing plant-based proteins as emulsifiers is also emerging since plants are recognized as rich sources of protein, intended for different purposes, with low production costs and efficient extraction protocols. Constant improvement in their functional properties (emulsification, foamability, solubility, gelation, hydration, miscibility, aroma trapping, and viscosity) is leading to their further applications in the food and pharmaceutical industries.

## Figures and Tables

**Table 1:** Physical properties of oil-in-water emulsions containing rosemary/oregano extract with the addition of commercial pea protein as emulsifier. ^A,B^ The same superscript capital letters within a row denote no significant differences (*p* > 0.05) between the values obtained for the different emulsions under the same experimental conditions according to Tukey’s ANOVA. ^a–j^ The same superscript lowercase letters within a column denote no significant differences (*p* > 0.05) between the values obtained for the emulsions under the different experimental conditions according to Tukey’s ANOVA.

Sample	Zeta Potential (mV)		Conductivity (mS cm^−1^)	
	Rosemary	Oregano	Rosemary	Oregano
1	−43.22 ± 1.76 ^A,a^	−42.47 ± 2.73 ^A,a^	0.0063 ± 0.00 ^A,a^	0.0051 ± 0.00 ^B,a^
2	−49.91 ± 3.37 ^A,a,c^	−45.20 ± 0.95 ^A,a^	0.0067 ± 0.00 ^A,a,f^	0.0075 ± 0.00 ^A,b^
3	−51.14 ± 2.87 ^A,a,c^	−52.28 ± 1.35 ^A,b^	0.0099± 0.00 ^A,a,f^	0.0164 ± 0.00 ^B,c^
4	−47.07 ± 1.98 ^A,a^	−48.65 ± 2.78 ^A,a^	0.0104 ± 0.00 ^A,a,f^	0.0112 ± 0.00 ^A,b^
5	−48.32 ± 1.41 ^A,a^	−43.09 ± 2.45 ^A,a^	0.0117 ± 0.00 ^A,b,f^	0.0093 ± 0.00 ^A,b^
6	−41.37 ± 4.50 ^A,a^	−46.97 ± 3.76 ^A,a^	0.0103 ± 0.00 ^A,a,f^	0.0499 ± 0.00 ^B,a^
7	−51.77 ± 0.67 ^A,a,c^	−37.71 ± 2.31 ^B,a^	0.0301 ± 0.00 ^A,c^	0.0099 ± 0.00 ^B,b^
8	−67.22 ± 2.24 ^A,b,c^	−57.18 ± 4.47 _B,c_	0.0453 ± 0.00 ^A,d^	0.0286 ± 0.00 ^B,d^
9	−39.53 ± 0.26 ^A,a,c^	−51.39 ± 3.53 ^B,a,c^	0.0234 ± 0.00 ^A,e,i^	0.0064 ± 0.00 ^B,b^
10	−46.29 ± 5.87 ^A,a,b^	−43.94 ± 1.73 ^A,a,d^	0.0114 ± 0.00 ^A,f^	0.0177 ± 0.00 ^B,c^
11	−40.50 ± 1.84 ^A,a^	−50.41 ± 1.20 ^B,a,c^	0.0043 ± 0.00 ^A,g,d^	0.0052 ± 0.00 ^B,b^
12	−38.52 ± 3.28 ^A,a^	−45.60 ± 3.98 ^A,a^	0.0092 ± 0.00 ^A,a,f^	0.0168 ± 0.00 ^B,c^
13	−49.67 ± 2.88 ^A,a^	−52.21 ± 1.92 ^A,d,c^	0.0223 ± 0.00 ^A,h,e,i^	0.0098 ± 0.00 ^B,b^
14	−48.02 ± 2.95 ^A,a^	−56.92 ± 1.22 ^A,e,c^	0.0251 ± 0.00 ^A,i,b^	0.0179 ± 0.00 ^B,c^
15	−45.20 ± 2.16 ^A,a^	−51.54 ± 3.50 ^A,f,c^	0.0072 ± 0.00 ^A,a,f^	0.0099 ± 0.00 ^A,b^
16	−46.11 ± 0.60 ^A,a^	−56.24 ± 2.48 ^B,g,c^	0.0148 ± 0.00 ^A,j,f^	0.0128 ± 0.00 ^A,c,b^
17	−42.95 ± 3.73 ^A,a^	−53.32 ± 0.30 ^B,h,c^	0.0098 ± 0.00 ^A,a,b,f^	0.0094 ± 0.00 ^A,b^

**Table 2:** Chemical properties of oil-in-water emulsions containing rosemary/oregano extract with the addition of commercial pea protein as emulsifier. ^A,B^ The same superscript capital letters within a row denote no significant differences (*p* > 0.05) between the values obtained for the different emulsions at the same experimental conditions according to Tukey’s ANOVA. ^a–j^ The same superscript lowercase letters within a column denote no significant differences (*p* > 0.05) between values obtained for emulsions prepared at different experimental conditions according to Tukey’s ANOVA.

Sample	TPC (mg_GAE_ g_dw_^−1^)		DPPH (mmol_Trolox_ g_dw_^−1^)		FRAP (mmol_FeSO4·7H2O_ g_dw_^−1^)	
	Rosemary	Oregano	Rosemary	Oregano	Rosemary	Oregano
1	7.01 ± 0.00 ^A,a^	15.02 ±2.36 ^B,a^	0.08 ± 0.01 ^A,a^	0.64 ± 0.03 ^B,a^	0.13 ± 0.01 ^A,a^	0.06 ± 0.01 ^A,a^
2	1.35 ± 1.14 ^A,a^	9.18 ± 1.18 ^B,a,b^	0.32 ± 0.03 ^A,a^	0.41 ± 0.02 ^A,a^	0.12 ± 0.00 ^A,a^	0.15 ± 0.02 ^A,a^
3	50.97 ± 0.38 ^A,b^	113.79 ± 1.90 ^B,c^	0.30 ± 0.03 ^A,a^	0.78 ± 0.01 ^B,a,b^	0.49 ± 0.03 ^A,c^	0.71 ± 0.11 ^B,b,c^
4	33.71 ± 1.14 ^A,c^	111.28 ± 2.40 ^B,c,d^	0.13 ± 0.00 ^A,a^	0.56 ± 0.05 ^B,a,b^	0.22 ± 0.03 ^A,a^	0.62 ± 0.00 ^B,b,c^
5	37.48 ± 0.38 ^A,c^	54.25 ± 1.18 B^,e^	0.17 ± 0.01 ^A,a^	0.40 ± 0.04 ^A,a,b^	0.16 ± 0.01 ^A,a,g^	0.43 ± 0.02 ^B,d,e^
6	25.35 ± 3.05 ^A,d^	53.97 ± 0.79 ^B,e^	0.18 ± 0.02 ^A,a^	0.30 ± 0.03 ^A,b,c^	0.13 ± 0.03 ^A,a^	0.14 ± 0.15 ^A,a^
7	28.04 ± 1.53 ^A,c,d^	55.92 ± 0.39 ^B,e^	0.22 ± 0.05 ^A,a^	0.80 ± 0.01 ^B,a^	0.15 ± 0.02 ^A,a^	0.18 ± 0.01 ^A,a^
8	32.63 ± 3.43 ^A,c,d^	56.20 ± 1.57 ^B,e,f^	0.19 ± 0.03 ^A,a^	0.83 ± 0.06 ^B,a^	0.13 ± 0.04 ^A,a^	0.33 ± 0.02 ^B,d,e^
9	13.75 ± 7.25 ^A,a,e^	9.74 ± 0.39 ^A,b,g,h^	0.07 ± 0.04 ^A,a,b^	0.66 ± 0.01 ^B,a,b^	0.07 ± 0.01 ^A,a,d^	0.16 ± 0.01 ^A,a^
10	53.93 ± 0.76 ^A,b^	105.16 ± 1.60 ^B,d,h^	0.33 ± 0.01 ^A,a^	0.60 ± 0.00 ^A,a,b^	0.26 ± 0.01 ^A,a,b^	1.01 ± 0.08 ^B,f^
11	3.51 ± 1.14 ^A,a^	6.68 ± 0.00 ^A,b,f^	0.06 ± 0.01 ^A,a,c^	0.66 ± 0.03 ^B,a,b^	0.09 ± 0.00 ^A,a,e^	0.07 ± 0.00 ^A,a^
12	45.57 ± 2.67 ^A,b^	107.39 ± 6.30 ^B,c,e,g^	0.35 ± 0.04 ^A,a,d^	0.67 ± 0.04 ^B,a,b^	0.24 ± 0.01 ^A,a^	0.99 ± 0.04 ^B,f^
13	22.92 ± 3.43 ^A,d^	54.81 ± 0.39 ^B,e^	0.18 ± 0.00 ^A,a^	0.31 ± 0.06 ^A,b,c^	0.32 ± 0.00 ^A,f^	0.51 ± 0.00 ^B,c,d^
14	22.65 ± 0.76 ^A,d^	57.03 ± 1.18 ^B,e^	0.18 ± 0.06 ^A,a^	0.34 ± 0.03 ^A,b,c^	0.29 ± 0.00 ^A,g^	0.48 ± 0.08 ^B,b,d^
15	17.53 ± 2.67 ^A,e^	52.58 ± 2.75 ^B,e,i^	0.20 ± 0.01 ^A,a^	0.32 ± 0.00 ^A,b,c^	0.30 ± 0.01 ^A,h^	0.65 ± 0.05 ^B,b,c^
16	15.64 ± 1.53 ^A,e^	56.48 ± 1.97 ^B,e^	0.20 ± 0.04 ^A,a^	0.35 ± 0.04 ^A,b,c^	0.31 ± 0.01 ^A,i^	0.55 ± 0.07 ^B,c,d^
17	14.02 ± 0.76 ^A,a,e^	45.90 ± 1.18 ^B,i^	0.23 ± 0.02 ^A,a^	0.69 ± 0.46 ^B,a^	0.13 ± 0.01 ^A,a,j^	0.36 ± 0.00 ^B,d,e^

**Table 3:** Response surface models regression coefficients and determination coefficients for chemical properties of oil-in-water emulsions containing rosemary extract with the addition of commercial pea protein as an emulsifier (bold values: *p* < 0.05).

Analyzed Property	Input Variable	Intercept ± Standard Error	Regression Coefficients (L-Linear, Q-Quadratic) ± Standard Error	*p*	R^2^	R^2^_adj._
TPC (mg_GAE_ g_dw_^−1^)	Oil concentration (%)	27.647 ± 2.355	−3.809 ± 1.725 (L) −2.232 ± 1.223 (Q)	0.052 (L) 0.098 (Q)	0.94	0.90
	Emulsifier concentration (%)		**19.197 ± 2.644 (L)** 0.623 ± 2.147 (Q)	**0.000 (L)** 0.778 (Q)		
	Rotational speed (rpm)		−2.595 ± 1.725(L) **−4.564 ± 1.189(Q)**	0.163(L) **0.003(Q**)		
DPPH (mmol_Trolox_ g_dw_^−1^)	Oil concentration (%)	0.205 ± 0.043	0.006± 0.032 (L) 0.002 ± 0.022 (Q)	0.857 (L) 0.932 (Q)	0.35	0.00
	Emulsifier concentration (%)		0.075 ± 0.048 (L) −0.003 ± 0.039 (Q)	0.155 (L) 0.940 (Q)		
	Rotational speed (rpm)		0.01 ± 0.032 (L) 0.002 ± 0.022(Q)	0.766 (L) 0.908 (Q)		
FRAP (mmol_FeSO4·7H2O_ g_dw_^−1^)	Oil concentration (%)	0.142 ± 0.041	−0.041 ± 0.030 (L) 0.030 ± 0.021(Q)	0.199 (L) 0.187 (Q)	0.76	0.62
	Emulsifier concentration (%)		0.045 ± 0.046 (L) 0.056 ± 0.037 (Q)	0.354 (L) 0.165 (Q)		
	Rotational speed (rpm)		−0.0008 ± 0.030 (L) **0.074 ± 0.021 (Q)**	0.979 (L) **0.005(Q)**		

**Table 4:** Response surface models regression coefficients and determination coefficients for chemical properties of oil-in-water emulsions containing oregano extract with the addition of commercial pea protein as emulsifier (bold values: *p* < 0.05).

Analyzed Property	Input Variable	Intercept ± Standard Error	Regression Coefficients (L-Linear, Q-Quadratic) ± Standard Error	*p*	R^2^	R^2^_adj._
TPC (mg_GAE_ g_dw_^−1^)	Oil concentration (%)	59.375 ± 0.978	−1.043 ± 0.708 (L) **−1.408 ± 0.515(Q)**	0.175 (L) **0.023(Q)**	0.99	0.99
	Emulsifier concentration (%)		**48.826 ± 1.104 (L)** 0.800 ± 0.905 (Q)	**0.000 (L)** 0.399 (Q)		
	Rotational speed (rpm)		0.38254 ± 0.708542 (L) **1.304 ± 0.501 (Q)**	0.60 (L) **0.029 (Q)**		
DPPH (mmol_Trolox_ g_dw_^−1^)	Oil concentration (%)	0.706 ± 0.059	−0.065 ± 0.043 (L) −0.062 ± 0.031 (Q)	0.164 (L) 0.079 (Q)	0.76	0.60
	Emulsifier concentration (%)		**0.176 ± 0.067 (L)** **−0.145 ± 0.055 (Q)**	**0.028 (L)** **0.026 (Q)**		
	Rotational speed (rpm)		**0.126 ± 0.043 (L)** **−0.076 ± 0.030 (Q)**	**0.016 (L)** **0.033 (Q)**		
FRAP (mmol_FeSO4·7H2O_ g_dw_^−1^)	Oil concentration (%)	0.430 ± 0.059	−0.018 ± 0.043 (L) **0.109 ± 0.031 (Q)**	0.687 (L) **0.006 (Q)**	0.91	0.84
	Emulsifier concentration (%)		**0.380 ± 0.066 (L)** −0.019 ± 0.054 (Q)	**0.0003 (L)** 0.731 (Q)		
	Rotational speed (rpm)		−0.020 ± 0.043 (L) 0.027 ± 0.030 (Q)	0.644 (L) 0.400 (Q)		

**Table 5:** Physical properties of rosemary/oregano extracts and oil-in-water emulsions containing rosemary/oregano extract, with the addition of commercial pea protein as emulsifier, before and during in vitro digestion. ^A,B^ The same superscript capital letters within a row denote no significant differences (*p* > 0.05) between the values obtained for the different plant aqueous extracts and emulsions at the same experimental conditions according to Tukey’s ANOVA. ^a–d^ The same superscript lowercase letters within a column denote no significant differences (*p* > 0.05) between values during in vitro digestion according to Tukey’s ANOVA.

Sample	Total Dissolved Solids (g L^−1^)		Conductivity (mS cm^−1^)	
	Rosemary	Oregano	Rosemary	Oregano
Plant extract (initial)	0.09 ± 0.00 ^A,a^	0.31 ± 0.01 ^B,a^	0.18 ± 0.00 ^A,a^	0.63 ± 0.00 ^B,a^
Plant extract (mouth)	2.46 ± 0.02 ^A,b^	2.42 ± 0.06 ^A,b^	4.93 ± 0.03 ^A,b^	4.90 ± 0.08 ^A,b^
Plant extract (stomach)	1.25 ± 0.04 ^A,c^	1.35 ± 0.02 ^A,c^	2.50 ± 0.01 ^A,c^	2.61 ± 0.15 ^A,c^
Plant extract (duodenum)	4.28 ± 0.04 ^A,d^	4.88 ± 0.05 ^A,d^	8.39 ± 0.21 ^A,d^	9.80 ± 0.06 ^B,d^
Emulsion (initial)	0.02 ±0.00 ^A,a^	0.04 ±0.00 ^A,a^	0.04 ± 0.00 ^A,a^	0.01 ± 0.00 ^B,a^
Emulsion (mouth)	1.18 ± 0.03 ^A,b^	2.36 ± 0.15 ^B,b^	2.39 ± 0.02 ^A,b^	4.92 ± 0.08 ^B,b^
Emulsion (stomach)	1.61 ± 0.04 ^A,c^	1.23 ± 0.01 ^B,c^	3.24 ± 0.06 ^A,c^	2.46 ± 0.01 ^B,c^
Emulsion (duodenum)	4.69 ± 0.08 ^A,d^	4.93 ± 0.00 ^B,d^	9.48 ± 0.06 ^A,d^	9.85 ± 0.04 ^B,d^

**Table 6:** Bioavailability of total polyphenols from rosemary/oregano extracts and oil-in-water emulsions containing rosemary/oregano extract after in vitro digestion, with the addition of commercial pea protein as emulsifier.

		Rosemary	Oregano
TPC (mg_GAE_ g_dw_^−1^)	Plant extract	5.09%	5.99%
	Emulsified plant extract	67.78%	3.39%
DPPH (mmol_Trolox_ g_dw_^−1^)	Plant extract	42.11%	17.07%
	Emulsified plant extract	69.23%	76.47%
FRAP (mmol_FeSO4·7H2O_ g_dw_^−1^)	Plant extract	16.39%	5.07%
	Emulsified plant extract	14.29%	5.36%

**Table 7:** Box–Behnken experimental design for emulsification process.

Experiment No.	Oil Concentration in Emulsified Plant Extracts (% *w*/*w*)	Emulsifier Concentration (% *w*/*w*)	Rotational Speed (rpm)
1	10 (−1)	0.10 (−1)	25,000 (0)
2	25 (+1)	0.10 (−1)	25,000 (0)
3	10 (−1)	1.00 (+1)	25,000 (0)
4	25 (+1)	1.00 (+1)	25,000 (0)
5	10 (−1)	0.50 (0)	15,000 (−1)
6	25 (+1)	0.50 (0)	15,000 (−1)
7	10 (−1)	0.50 (0)	35,000 (+1)
8	25 (+1)	0.50 (0)	35,000 (+1)
9	15 (0)	0.10 (−1)	15,000 (−1)
10	15 (0)	1.00 (+1)	15,000 (−1)
11	15 (0)	0.10 (−1)	35,000 (+1)
12	15 (0)	1.00 (+1)	35,000 (+1)
13	15 (0)	0.50 (0)	25,000 (0)
14	15 (0)	0.50 (0)	25,000 (0)
15	15 (0)	0.50 (0)	25,000 (0)
16	15 (0)	0.50 (0)	25,000 (0)
17	15 (0)	0.50 (0)	25,000 (0)

## Data Availability

Not applicable.
